# Remote Patient Monitoring and Machine Learning in Acute Exacerbations of Chronic Obstructive Pulmonary Disease: Dual Systematic Literature Review and Narrative Synthesis

**DOI:** 10.2196/52143

**Published:** 2024-09-09

**Authors:** Henry Mark Granger Glyde, Caitlin Morgan, Tom M A Wilkinson, Ian T Nabney, James W Dodd

**Affiliations:** 1 EPSRC Centre for Doctoral Training in Digital Health and Care University of Bristol Bristol United Kingdom; 2 Academic Respiratory Unit, Translational Health Sciences Bristol Medical School University of Bristol Bristol United Kingdom; 3 Clinical and Experimental Science University of Southampton Southampton United Kingdom; 4 School of Engineering and Mathematics University of Bristol Bristol United Kingdom

**Keywords:** acute exacerbations of COPD, chronic obstructive pulmonary disease, exacerbate, exacerbation, exacerbations, remote patient monitoring, RPM, predict, prediction, predictions, predictive, machine learning, monitoring, remote, COPD, pulmonary, respiratory, lung, lungs, literature review, literature reviews, synthesis, narrative review, narrative reviews, review methods, review methodology

## Abstract

**Background:**

Acute exacerbations of chronic obstructive pulmonary disease (AECOPD) are associated with high mortality, morbidity, and poor quality of life and constitute a substantial burden to patients and health care systems. New approaches to prevent or reduce the severity of AECOPD are urgently needed. Internationally, this has prompted increased interest in the potential of remote patient monitoring (RPM) and digital medicine. RPM refers to the direct transmission of patient-reported outcomes, physiological, and functional data, including heart rate, weight, blood pressure, oxygen saturation, physical activity, and lung function (spirometry), directly to health care professionals through automation, web-based data entry, or phone-based data entry. Machine learning has the potential to enhance RPM in chronic obstructive pulmonary disease by increasing the accuracy and precision of AECOPD prediction systems.

**Objective:**

This study aimed to conduct a dual systematic review. The first review focuses on randomized controlled trials where RPM was used as an intervention to treat or improve AECOPD. The second review examines studies that combined machine learning with RPM to predict AECOPD. We review the evidence and concepts behind RPM and machine learning and discuss the strengths, limitations, and clinical use of available systems. We have generated a list of recommendations needed to deliver patient and health care system benefits.

**Methods:**

A comprehensive search strategy, encompassing the Scopus and Web of Science databases, was used to identify relevant studies. A total of 2 independent reviewers (HMGG and CM) conducted study selection, data extraction, and quality assessment, with discrepancies resolved through consensus. Data synthesis involved evidence assessment using a Critical Appraisal Skills Programme checklist and a narrative synthesis. Reporting followed PRISMA (Preferred Reporting Items for Systematic Reviews and Meta-Analyses) guidelines.

**Results:**

These narrative syntheses suggest that 57% (16/28) of the randomized controlled trials for RPM interventions fail to achieve the required level of evidence for better outcomes in AECOPD. However, the integration of machine learning into RPM demonstrates promise for increasing the predictive accuracy of AECOPD and, therefore, early intervention.

**Conclusions:**

This review suggests a transition toward the integration of machine learning into RPM for predicting AECOPD. We discuss particular RPM indices that have the potential to improve AECOPD prediction and highlight research gaps concerning patient factors and the maintained adoption of RPM. Furthermore, we emphasize the importance of a more comprehensive examination of patient and health care burdens associated with RPM, along with the development of practical solutions.

## Introduction

Chronic obstructive pulmonary disease (COPD) is a disease defined by airway obstruction, airway inflammation, and in some cases parenchymal destruction (emphysema). COPD accounts for 55% of all chronic respiratory diseases [1] and is characterized by intermittent periods of significantly worsening symptoms known as exacerbations [2]. After a severe exacerbation, the in-hospital mortality is 6.7% [3]. Subsequently, the average mortality rates at 3 and 6 months stand at 18% and 26%, respectively, with a notable 50% mortality rate observed at 3.6 years [3,4]. Exacerbations increase airway and systemic inflammation and disease progression and cause a reduction in quality of life [5-11]. It is estimated that exacerbations in COPD account for 45% of COPD-related costs [12]. Patients who experience frequent acute exacerbations of COPD (AECOPD) have more primary care interactions, increased emergency department (ED) presentations, increased hospitalizations, and increased admissions to the intensive care unit [13]. A recent research priority-setting partnership in COPD found the highest-rated issue by patients or carers to be “identify better ways to prevent exacerbations” [14]. The researchers highlighted the importance of predicting and preventing exacerbations.

There is evidence to suggest that reducing delays in treatment and correct identification of exacerbations can reduce the severity of exacerbations, improve health-related quality of life (HRQoL), and reduce recovery time after an exacerbation. Wilkinson et al [15] found that a longer time to treatment in AECOPD was associated with an increase in the recovery time of exacerbation symptoms [15]. Moreover, they demonstrated that a greater number of correctly identified exacerbations treated by a physician resulted in a better HRQoL, as seen in the lower total St George’s Respiratory Questionnaire scores.

Remote patient monitoring (RPM) is a method of health care delivery that uses wearable devices and sensors to gather patient data outside of traditional health care settings. Using RPM, data that may provide a more detailed picture of the patient’s health become available. The patient, along with a team of health care professionals, can review these data to promptly identify changes in the patient’s health status, enabling early detection of potential exacerbations and facilitating timely intervention. Nevertheless, the current role of RPM in managing AECOPD remains uncertain. While some studies indicate potential benefits, others show no significant effects. Therefore, conducting a systematic review and synthesizing existing evidence becomes crucial to comprehend the current state of the art. This assessment is essential for defining the next steps in the development and testing of technology to address this global health challenge. Notably, leveraging machine learning approaches on the gathered data holds promise in enhancing RPM’s predictive capabilities. Thus, a comprehensive review is imperative to assess the current progress in this field.

In this dual systematic review, we aim to identify various approaches to remote monitoring for exacerbation intervention and prediction in AECOPD. We combine insights from both machine learning and remote clinical monitoring perspectives. The first review focuses on randomized controlled trials (RCTs) using RPM as an intervention to treat or improve AECOPD. The second review investigates studies integrating machine learning with RPM to predict AECOPD. This comprehensive approach enables us to provide a novel understanding of digitally enabled AECOPD interventions. We review the evidence and concepts behind RPM and machine learning; discuss the strengths, limitations, and clinical applications of available systems; and generate recommendations to enhance patient and health care system outcomes.

## Methods

We conducted 2 systematic literature searches in accordance with the PRISMA (Preferred Reporting Items for Systematic Reviews and Meta-Analyses; Multimedia Appendix 1) statement [16].

### Search Strategy

The searches were conducted between April and May 2023 in 2 electronic databases (Scopus and Web of Science) covering publications since the databases began. For Scopus, this includes records dating back to 1788, and for Web of Science, the database covers literature dating back to the early 1900s. The first search strategy included search strings in 4 main areas: COPD, RPM (telemedicine, telemonitoring, RPM, real-time monitoring, telehealth, mobile health, and digital health), study design (intervention and trial), and outcome (exacerbation frequency, exacerbation duration, ED presentations, hospital admissions, hospital readmissions, primary care interaction, health care costs, quality of life, and days in hospital). The second search strategy also included COPD and RPM but did not include study design, and instead of outcome, the search term was machine learning modeling (machine learning, deep learning, prediction models, and algorithms). The full search strategies for each database are presented in the Multimedia Appendix 2. Articles published in peer-reviewed journals or conference proceedings were considered for review. We did not include abstracts, dissertations, systematic reviews, or case studies.

### Study Selection

To be included in the first search, studies were required to (1) specifically examine the use of RPM in COPD; (2) be an RCT; (3) have an exacerbation-related outcome variable, that is, hospital admissions, exacerbation frequency, and HRQoL; (4) be published between the start date of each electronic database and May 2023; (5) be full freely available articles; and (6) be published in English.

For the second search, fewer studies were available. Therefore, studies were not required to be an RCT, and instead of including an exacerbation-related outcome variable, studies were required to incorporate a form of artificial intelligence modeling, usually machine learning algorithms, for exacerbation prediction.

Papers were excluded from the study for any one of the following reasons: (1) the study was a systematic literature review, (2) the study did not include any one outcome related to either the first search or the second search, (3) the focus of the intervention was behavior change (physical activity, medication adherence, and inhaler technique) or remote rehabilitation (usually pulmonary rehabilitation) rather than remote monitoring, and (4) the main study outcome was cost and did not include patient-related outcomes.

Machine learning studies were intentionally excluded from the first search. This decision was guided by the unique emphasis of each search: the first centered on RPM with clinical monitoring, while the second focused on RPM with an emphasis on machine learning.

Two authors (HMGG and CM) independently assessed the results obtained from the first literature search. Articles were screened in 4 steps: first, duplicates were removed, and then the title, abstract, and keywords were screened. Articles were screened on the inclusion and exclusion criteria outlined above. If authors were unable to determine the suitability during the screening, full-text articles were accessed for inclusion criteria and exclusion criteria. Full-text articles were excluded for not reporting outcomes for patients with COPD or the accuracy of COPD exacerbation prediction separately (in the case of studies with multiple diseases).

Remote monitoring studies were not included in the review if they reported on remote monitoring as an alternative to hospitalization for exacerbation treatment, did not include the specifics of the RPM use, or focused on the diagnosis of AECOPD rather than prediction.

### Evidence Assessment and Narrative Synthesis

**Figure 1:**
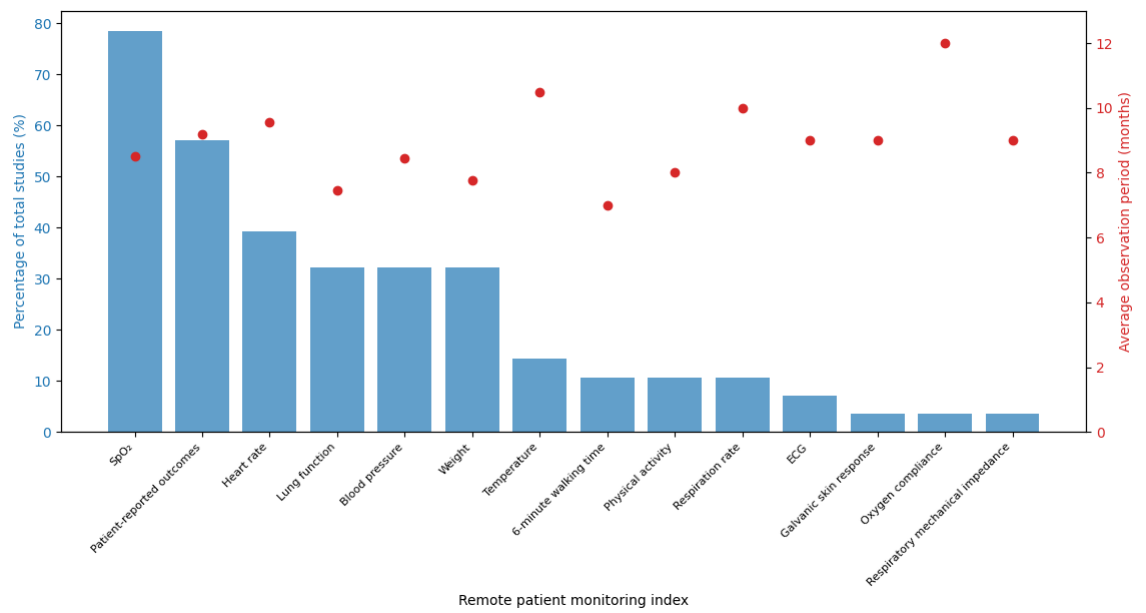
Distribution of remote patient monitoring indices and average study duration for the first search. The figure illustrates the percentage of total studies each remote patient monitoring index appears in, alongside the average duration of these studies. ECG: electrocardiogram; SpO_2_: oxygen saturation.

**Figure 2:**
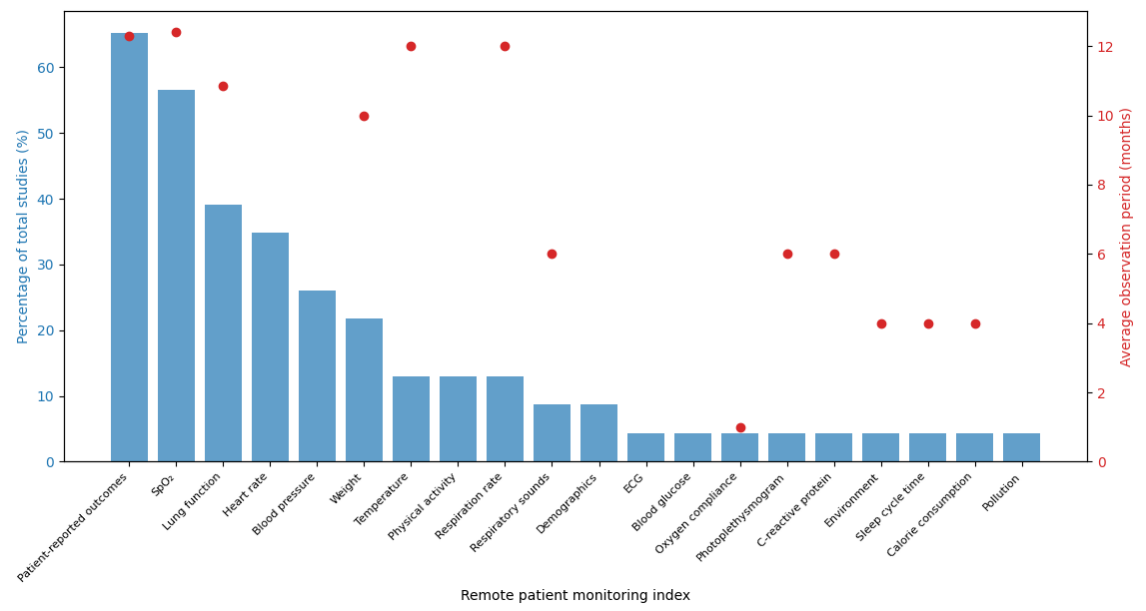
Distribution of remote patient monitoring indices and average study duration for the second search. The figure illustrates the percentage of total studies each remote patient monitoring index appears in, alongside the average duration of these studies. ECG: electrocardiogram; SpO_2_: oxygen saturation.

In the process of narrative synthesis, the initial step involved the identification and documentation of comparator groups. The lead author (HMGG) identified and documented comparator groups, capturing patient numbers, age, sex, and forced expiratory volume in 1 second (FEV1). Subsequently, the lead author recorded the specific RPM indices used in these studies, providing insight into the monitored parameters. These results are detailed in Multimedia Appendix 3. The RPM index, including its percentage occurrence in studies and the average study duration, can be found in Figures 1 and 2. The lead author identified and described the method of detection or prediction of AECOPD. This encompasses either a flagging system or clinical oversight or the application of machine learning approaches. Finally, the outcomes were described, documenting key measures, such as hospitalizations, HRQoL, and performance metrics, like sensitivity and accuracy. The results from the narrative synthesis can be found in Multimedia Appendix 3, which the remaining authors (ITN, JWD, and TMAW) checked.

**Figure 3:**
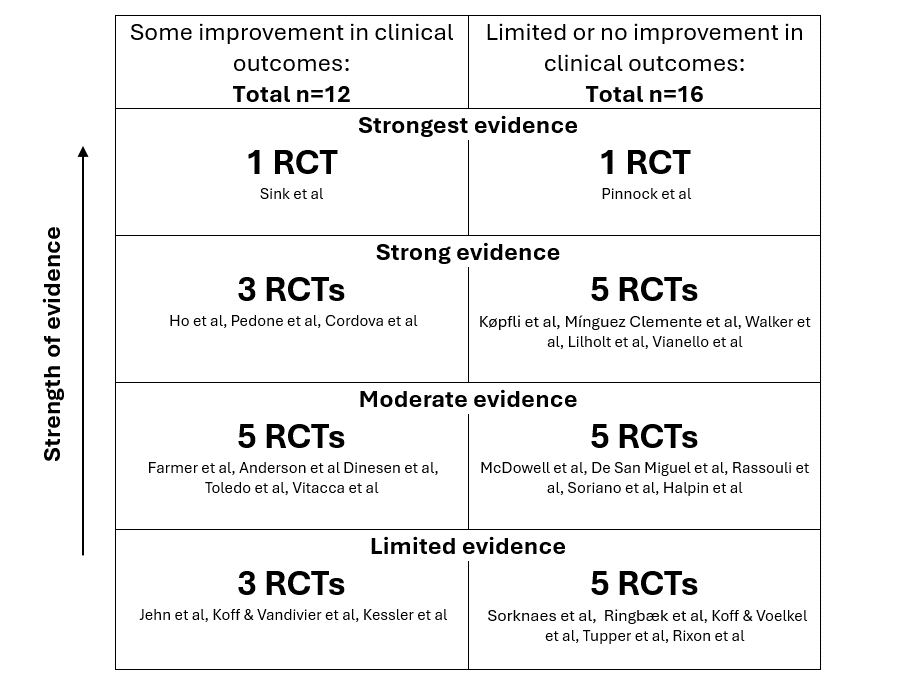
Rankings of RCTs on remote patient monitoring in chronic obstructive pulmonary disease according to the Critical Appraisal Skills Programme RCT checklist. RCT: randomized controlled trial.

For the initial search, we use the Critical Appraisal Skills Programme (CASP) RCT checklist. This tool is designed to systematically assess the validity, results, and relevance of RCTs, helping us gauge the quality of the evidence. Questions 9 and 11 were omitted from the evaluation as they are not relevant to this assessment. Studies satisfying 90% (9/10) of the criteria were designated as having the highest level of evidence (strongest evidence). Those meeting 80% (8/10) were categorized as strong evidence, while those meeting 70% (7/10) were classified as moderate evidence. Studies fulfilling 60% (6/10) of the criteria were considered to have limited evidence. For the second search, we used the CASP cohort checklist to assess cohort study quality. Questions 7 and 12 were excluded due to their lack of relevance. As in the first search, studies meeting 90% (9/10), 80% (8/10), 70% (7/10), and 60% (6/10) of the criteria were categorized as strongest, strong, moderate, and limited quality, respectively. The resulting rankings are visualized in Figure 3 [17-44] and described in the *Results* section.

## Results

### Overview

**Figure 4:**
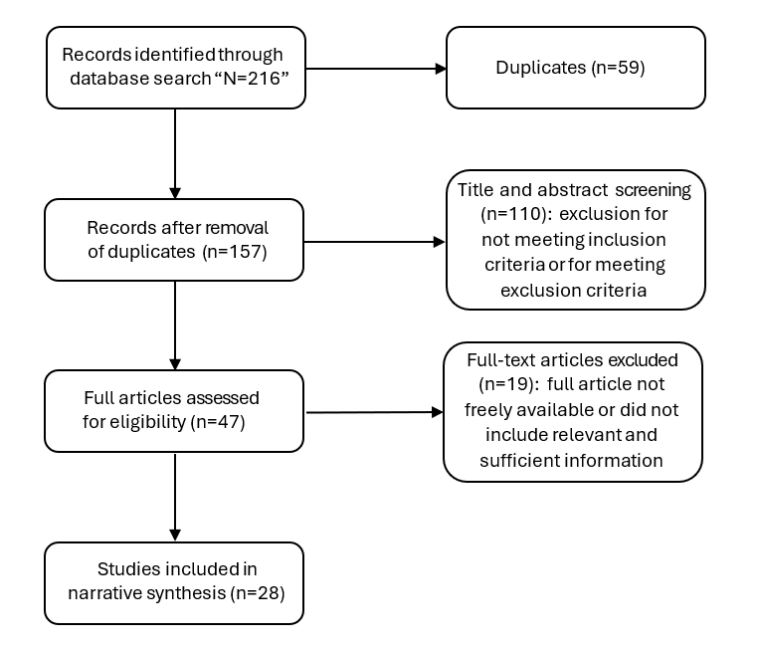
PRISMA (Preferred Reporting Items for Systematic Reviews and Meta-Analyses) flow diagram for the search procedure for randomized controlled trials where remote patient monitoring was used as an intervention to treat or improve acute exacerbations of chronic obstructive pulmonary disease.

We screened and analyzed data from April to May 2023. Through the first systematic search, we identified 216 studies, extending from 1998 to 2023. Of these, 28 were included in the review [17-44] (Figure 4).

To identify the distribution and duration of studies and RPM indices identified in our first search, we visualized the data using a dual-axis plot (Figure 2). On the x-axis, each RPM index is represented. The left y-axis (blue bar chart) indicates the percentage of studies in which each index appears, while the right y-axis (red points) shows the average duration of these studies. This visualization facilitates a comprehensive comparison between the prevalence and the duration of the studies associated with each RPM index.

We constructed a figure to present the outcomes and rankings derived from the CASP RCT checklist evaluation of the RCTs identified in our first search (Figure 3). The figure is divided into 2 columns, where the left column displays RCTs showing some improvement in clinical outcomes and the right column shows RCTs with limited or no improvement. Within each column, studies are ranked by the strength of evidence, from limited evidence at the bottom to the strongest evidence at the top. Each section indicating evidence strength includes the number of RCTs, with the specific studies cited below this label.

A detailed breakdown of the RCT characteristics, method of intervention delivery, and outcomes are available in Multimedia Appendix 3.

**Figure 5:**
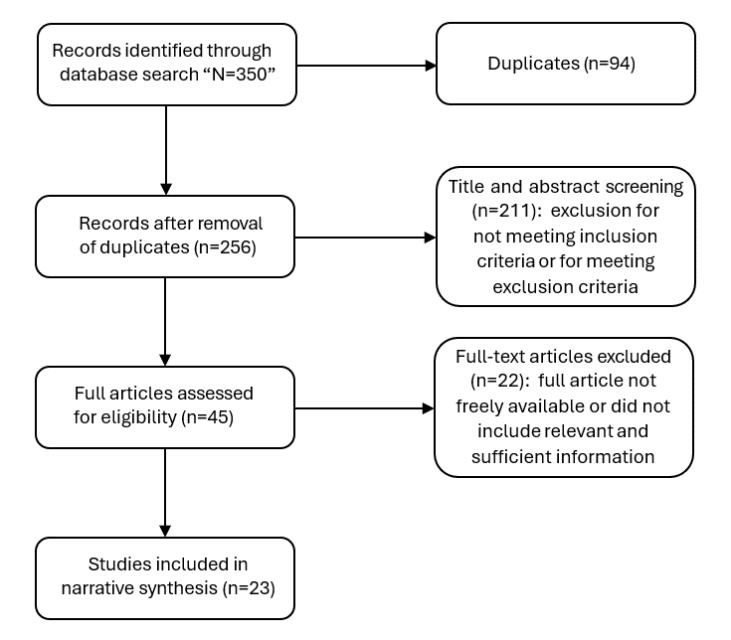
PRISMA (Preferred Reporting Items for Systematic Reviews and Meta-Analyses) flow diagram for the search procedure for empirical studies on remote patient monitoring and machine learning to predict acute exacerbations of chronic obstructive pulmonary disease.

The second systematic literature review search identified 350 articles, extending from 2005 to 2023. Of these, 23 were included in the review [45-68] (Figure 5).

To identify the distribution and duration of studies and RPM indices identified in our second search, we visualized the data using a dual-axis plot (Figure 2). On the x-axis, each RPM index is represented. The left y-axis (blue bar chart) indicates the percentage of studies in which each index appears, whereas the right y-axis (red points) shows the average duration of these studies. This visualization facilitates a comprehensive comparison between the prevalence and the duration of the studies associated with each RPM index.

The studies identified in the second search were ranked according to the CASP cohort checklist. The ranking is categorized by the quality of the study as follows: strongest (studies [52,58-61]), strong (studies [51,54,55,57,64-68]), moderate (studies [48-50,53,56]), and limited (studies [45-47,62,63]). This categorization helps in clearly delineating the quality across the identified studies. A detailed breakdown of the machine learning study characteristics, method of intervention delivery, and outcomes are available in the Multimedia Appendix 3.

### RPM for Intervention or Management of AECOPD

Our analysis revealed a tendency among RCTs toward nonimprovement or demonstrated a lack of statistical significance in indicators of improvement in exacerbation management (Figure 3).

Systematic reviews by both Jang et al [69] and Kruse et al [70] identified that RPM often does not show a significant improvement in patient outcomes. This may stem from the diversity of study approaches, as emphasized by Kruse et al [70]: “High variability between the articles and the ways they provided telemonitoring services created conflicting results from the literature review.” The varied study designs make comparisons challenging and likely contribute to the diverse reported success levels in the literature. Furthermore, the burden of frequent monitoring may impact the effectiveness of RPM, as the commonly used approaches are often resource-intensive and burdensome for both patients and clinicians, thereby hindering widespread adoption.

### RPM and Machine Learning for AECOPD Prediction

Machine learning, a subset of artificial intelligence, centers on leveraging data and algorithms to enable mathematical models for learning without direct instruction. In the context of predicting AECOPD, most studies using machine learning concentrate on assessing accuracy and related performance metrics based on retrospective data. This stands in contrast to efforts using RPM exclusively, which primarily aim at identifying improvements in patient outcomes through RCT. This makes the comparison of these 2 interrelated fields of research challenging, especially with regard to the indication of improving health outcomes for patients. Nevertheless, the narrative synthesis revealed that machine learning in conjunction with RPM facilitates highly accurate AECOPD prediction (Multimedia Appendix 3).

Orchard et al [59] compared machine learning with traditional symptom-counting algorithms in RPM for COPD exacerbation risk [59]. They found that machine learning “outperforms existing predictive algorithms.” On a data set from 135 patients over 363 days, basic symptom-counting algorithms had limited accuracy (area under the receiver operating characteristic curve [AUROC] of 0.60 and 0.58). Testing in real-world scenarios reduced the algorithms’ performance to no better than random decision-making. Machine learning models achieved the best AUROC of 0.74, exposing traditional algorithms’ notable shortcomings, including low accuracy and frequent false positives. This highlights machine learning’s potential to significantly enhance RPM accuracy in identifying and predicting COPD exacerbations.

So far, 2 patient-facing studies have been completed using machine learning for AECOPD prediction and intervention. The first tool evaluated was Adaptive Computerized COPD Exacerbation Self-management Support (ACCESS) [61], which demonstrated 97.4% sensitivity, but lower specificity at 65.6%. In an RCT, ACCESS did not significantly impact weeks without exacerbations or hospital admissions [61]. The second tool evaluated was the COPD Predict app that used patient-reported outcomes (PRO), FEV1, and C-reactive protein (CRP), predicting AECOPD with 97.9% sensitivity and 84.0% specificity [65]. For 6 months, COPD Predict reduced hospitalizations by 98%, but lower specificity resulted in 458 false positives, potentially hindering implementation. Using blood CRP levels for diagnosis and prediction may be impractical in real-world scenarios due to cost and access, indicating the need for the development of low-cost at-home biomarker sensors. COPD Predict identified exacerbations earlier than clinician-defined episodes, while ACCESS required major symptoms for 2 consecutive days, potentially delaying timely intervention. Furthermore, the low positive predictive value of access may have affected the trust of the patient-user and reduced the intervention’s effectiveness.

### Machine Learning Approaches

A total of 5 papers used neural networks for AECOPD prediction [48,50,58,59,68]. Neural networks excel at classification and prediction tasks due to their ability to model complex nonlinear relationships and automatically learn relevant features from data. Furthermore, neural networks can use transfer learning for efficient knowledge reuse, exhibit robustness to noisy data, and generalize well to new, unseen examples.

Nunavath et al [58] used a recurrent neural network (RNN), a subtype of artificial neural networks, for exacerbation prediction [58]. RNNs, commonly used in ordinal or temporal problems, generally demand substantial data for optimal performance. In this study, with a data set of 96 patients and approximately 7300 records collected over 2 years, data augmentation was applied to expand the training data, allowing the application of RNN, potentially resulting in a more potent, robust model for accuracy. van der Heijden et al [46], used Bayesian network algorithms to predict COPD exacerbations, offering advantages in incorporating previous knowledge, handling missing data through probabilistic inference, and providing interpretability by representing dependencies among variables. These models, known for their interpretability, are well-suited for dynamic environments like RPM. In studies by Fernandez-Granero et al [51,56], different machine learning methodologies were used in 2015 and 2017. The 2017 analysis, using a random forest (RF) classifier, demonstrated the benefits of ensemble learning and reduced overfitting compared with the support vector machine (SVM) model in 2015. This analysis also used the Markov chain Monte Carlo method for imputing missing data and feature subset selection, contributing to improved model performance. In addition, logistic regression was used by Shah et al [55] and Kronborg et al [57]. The models created in the study by Shah et al [55] encountered a notable trade-off between sensitivity and specificity (possibly stemming from challenges in data quality), and the model in the study by Kronborg et al [57] achieved an AUROC of 0.74, suggesting logistic regression limitations in capturing complex nonlinear relationships. Researchers should consider diverse approaches, acknowledging their strengths and limitations. Ensemble methods enhance robustness, Bayesian Networks offer interpretability, and techniques such as bootstrapping and data augmentation can improve data quality.

### Study Design

The studies identified in systematic searches exhibit heterogeneity in duration, sample size, and outcomes, influencing both RPM interventions and machine learning model development and testing.

Study durations vary from 3 months to 1 year, impacting AECOPD events due to seasonality. Machine learning interventions may face challenges in generalizing beyond specific months. Small studies with 10-30 participants may restrict the capture of exacerbations and hinder model generalizability to larger populations. While recruiting frequent exacerbators can help, it may lead to more false positives in less exacerbation-prone populations. Furthermore, smaller samples will limit the diversity of age, sex, ethnicity, socioeconomic status, disease severity, exacerbation frequency, and time since diagnosis. Diverse participants are crucial to validate intervention effectiveness.

Many studies use machine learning with data from interventional studies or RCTs to develop predictive models. The success of these studies is typically gauged by the accuracy and predictive capability of the generated models. While this is a crucial initial step to evaluate exacerbation prediction potential, relying solely on machine learning from RCT conditions may restrict applicability to real-world settings and diminish model accuracy. RCTs, conducted in controlled environments with specific criteria, might not fully capture real-world variability and complexity. Despite these difficulties, RCTs offer advantages, particularly in COPD, where a gold-standard diagnostic test for AECOPD is lacking. RCT data often involve rigorous testing, enhancing the certainty of accurately modeling true AECOPD events.

Defining and labeling exacerbations is a complex aspect of predicting AECOPD. It involves determining what qualifies as an exacerbation in the data, which presents significant challenges. Various methods exist for defining exacerbations, including patient-reported symptoms, clinician diagnosis, medication usage, hospitalization, or a combination of these criteria. Each approach has its advantages and drawbacks. Patient-reported symptoms and medication use are categorized as “symptom-based,” capturing all exacerbations efficiently but lacking oversight and verification with objective measures. Clinician diagnosis and hospitalization fall into the “event-based” category, with the former possibly being symptom-based if verified remotely. While clinician diagnosis is the gold standard, it can be resource-intensive. An alternative is to use hospitalization, which offers a clinician diagnosis, but may overlook milder events managed at home.

The challenge in labeling exacerbations lies in accurately distinguishing between exacerbation events and periods of stable health during machine learning model training. Failure to do so prevents the identification of the prodromal period and may lead to the misclassification of moments of symptom relief as stable health rather than ongoing exacerbations. To address this, an algorithmic approach is necessary, involving the delineation of data windows. Typically, these windows are created by capturing 14 days of exacerbation-free periods, a duration commonly recognized as the time it takes for an exacerbation to develop after the onset of symptoms. Failure to implement an effective label indicates an AECOPD prediction model may be ineffective in real-world applications.

### Patient Factors

Patient engagement and monitoring burden are crucial in RPM. Many studies in this review rely on the daily use of multiple sensors and PRO, yet still show high engagement. However, this may be influenced by the controlled research setting, providing extra attention and support, and motivating participants to engage. These studies often include rigorous monitoring and alerts for missed days. To assess real-world patient engagement, long-term data-gathering studies are often needed.

The burden of daily monitoring is frequently overlooked in interventions and model development studies, despite the requirement for high adherence. Concerns arise about time and effort, impacting daily life. Daily self-monitoring involves tracking symptoms, vital signs, medication adherence, and lifestyle factors, that potentially cause anxiety and stress. Some patients, with comorbidities or limited digital access, may struggle with devices or apps, leading to frustration and nonadherence. Patient and public involvement research is needed to understand user burden, develop strategies, and identify sensor types, minimizing burdens for increased long-term RPM adoption.

### A Review of Indices in RPM

The main types of data collected are physiological measures (blood pressure, heart rate [HR] or pulse rate [PR], respiratory rate [RR], weight, oxygen saturation [SpO_2_], and temperature), functional measures (lung function and physical activity [PA]), PRO (dyspnea, sputum, sleep quality, depression, anxiety, and HRQoL), self-report (physiological measures, medication-usage, exacerbation history, demographics, and medical history), and meteorological data.

### Physiological Measures

In a systematic review, Buekers et al [71] identified 71 papers measuring SpO_2_ in patients with COPD before exacerbations, revealing predictive limitations due to scant information on implementation and performance. Milkowska-Dymanowska et al [72] noted a significant SpO_2_ decrease before exacerbation, distinguishing it from systolic blood pressure, diastolic blood pressure, or HR. The findings by Shah et al [55] indicated that SpO_2_ (AUROC=0.658) outperformed PR (AUROC=0.578) in predicting exacerbations, highlighting SpO_2_’s superiority, while systolic blood pressure and HR and PR might offer some predictive capability. Mohktar et al [52] emphasized weight as an essential feature for AECOPD prediction. Dinesen et al [21] conducted an RCT that incorporated weight changes for clinical monitoring. Their intervention reduced hospitalization, affirming weight’s role in early AECOPD detection. van der Heijden et al [73] established a dependency between exacerbation and body temperature. RCT studies by Ho et al [30] and Pedone et al [24] reported positive patient outcomes using body temperature as an index for exacerbation monitoring. Shah et al [55] found that mean RR increased by 2 in the prodromal period leading to an exacerbation. However, a feasibility study by Chau et al [74], using RR to monitor patients, did not demonstrate improvements in patient outcomes, possibly due to inherent study design limitations. Burton et al [75] observed a mean SpO_2_ drop from 93.6% to 92.4% at exacerbation onset, and Shah et al [55] found a decrease from 94% to 93% in the prodromal period, enhancing AECOPD predictive models. Despite these findings, Burton et al [75] identified weak associations between physiological variables and exacerbation episodes, underscoring the need for improved algorithms or additional features for early event detection.

### Functional Measures

FEV1 serves as a crucial measure captured by a spirometer for diagnosing and monitoring obstructive lung diseases. Digital spirometry holds the potential to be a potent predictor of AECOPD. Limited data on lung function deterioration shortly before exacerbations exist, but Watz et al [76] through their post hoc analysis observed FEV1 decline 2 weeks before an exacerbation in the WISDOM (Withdrawal of Inhaled Steroids during Optimized Bronchodilator Management) clinical trial, highlighting spirometry’s use in identifying exacerbations. Patel et al [65] demonstrated spirometry’s predictive capability, achieving a sensitivity of 97.9% and specificity of 84.0% for AECOPD when combined with CRP and PRO.

PA, although infrequently used, offers a burdenless monitoring approach. Pedone et al [24], in their RCT, incorporating PA as a measure, showed a significant reduction in exacerbation events. Chawla et al [77] found that lower PA in the first week after discharge increased the likelihood of 30-day all-cause readmissions. Wrist-worn wearables measuring PA, prevalent through smartwatches, provide a useful, unobtrusive tool for monitoring for exacerbation prediction. While not a direct alternative to spirometry for lung function, they offer continuous monitoring without user engagement.

### PRO Measures

PRO are typically obtained through patient inputs into a digital diary, involving daily responses to yes-or-no or graded questions. Graded inquiries may include assessing chest tightness on a scale of 0 to 5. The rationale for incorporating PRO in RPM is robust. The chronic obstructive pulmonary disease assessment test (CAT), an 8-question validated tool [78], shows a positive correlation with COPD exacerbation risk [79]. A one-unit increase in CAT score signals an 8% higher risk of exacerbation [80]. Similar associations exist for other PRO measures, such as the Modified Medical Research Council dyspnea scale [11]. Most of the RCTs included in this review commonly use PRO as part of RPM. However, many of these studies do not show significant improvements in exacerbation-related outcomes. This might be attributed to the frequent use of nonvalidated, study-specific questionnaires for assessing PRO. To potentially enhance outcomes, a recommended shift would be from using nonvalidated questionnaires to prioritizing validated measures, such as the CAT.

### Biological Measures

Exacerbations in COPD correlate with various biomarkers, with CRP being extensively studied [81-84]. Patel et al [65] showcased CRP’s efficacy in COPD Predict, emphasizing its high sensitivity and specificity. However, limited CRP use in prediction models stems from challenges in deploying widespread systems with frequent point-of-care testing. Potential solutions include at-home finger-prick blood sampling, mail-in samples, or self-administered point-of-care tests, such as lateral flow tests used during the SARS-CoV-2 pandemic. Inflammatory cytokines in sputum and volatile organic compounds in exhaled breath are also targets for at-home monitoring, contingent on the development of suitable devices [85,86].

Despite the potential benefits, implementing remote biological monitoring encounters challenges in cost and scalability. Substantial expenses for developing and deploying home-based detection equipment, ensuring data accuracy, and managing sensor development, deployment, data transmission, and storage impede practical execution. Achieving scalability for costly digital biological monitoring technology demands a significant investment.

## Discussion

### Principal Findings

We have undertaken a systematic literature review and narrative synthesis of RPM and machine learning for COPD exacerbations to address the problem of identifying better ways to intervene early or prevent exacerbations to improve outcomes in AECOPD.

The narrative synthesis of available evidence reveals that RCTs using RPM to monitor patients at risk of AECOPD tend to exhibit nonsignificant outcomes. This can be attributed to the heterogeneity in study design and the use of traditional symptom-counting algorithms. In addition, there is limited insight into the accuracy or timeliness of AECOPD detection and intervention. Orchard et al [59] point to limitations relating to the basic algorithms that seem to generate alerts from RPM that may perform no better than chance [60] and highlights the potential of machine learning to significantly enhance the predictive capabilities of RPM.

We chose not to exclusively limit our second search to RCTs due to the lack of available RCTs in the relevant literature. The comparison of these 2 bodies of literature presents a challenge, and instead of considering them as comparators, it is more apt to view them as conjugates. While similarities exist in the methodologies of the 2 bodies of literature, particularly in the RPM indices, there are significant divergences in their outcomes. The first search primarily focuses on patient outcomes, whereas the second centers on the performance of machine learning models. This contrast underscores the importance of a shift in the trajectory of research: RCTs of RPM in AECOPD should introduce machine learning models to identify their efficacy and draw a conclusion to the question if early detection of AECOPD can improve patient outcomes. While early research for machine learning in the prediction of AECOPD is promising, there is much need for further development in the field. Much of the literature evaluates the ability of machine learning models to predict AECOPD yet fails to provide evidence on how these models translate into improving patient outcomes. Notable exceptions include COPD Predict developed by Patel et al [65], which may reduce hospitalization [66], but it requires frequent blood testing and the use of expensive home-based detection equipment. Due to the lack of research in this field, it is too early to determine if exacerbation prediction and intervention can be deployed with machine learning to improve AECOPD outcomes, especially without the incorporation of biological indices.

Some adjustments can be made in future studies that may lead to more robust conclusions. For example, exacerbation definition and labeling are heterogeneous and would benefit from standardization, it is critical to ensure that there is sufficient data capture by increasing the number of participants and keeping the study length to a minimum of 1 year should result in better generalization of predictive models. In addition, integrating data augmentation, resampling, and feature selection techniques can further enhance training data for machine learning. Furthermore, incorporating neural networks into a modeling approach may greatly enhance the predictive power of AECOPD models. Clinically validated PRO, like that of CAT, should be considered as an alternative to study-specific questionnaires. SpO_2_, RR, HR, weight, FEV1, and peak expiratory flow appear frequently in remotely monitored indices and may have the potential for exacerbation prediction if combined with machine learning. In addition, further research could demonstrate the predictive capabilities of physical activity. Patient and public involvement research needs to be conducted with these sensors to explore the feasibility and likelihood of long-term adherence. The incorporation of sensing technologies that monitor in the background and are seemingly burdenless (wrist-worn wearables or smartwatches) should be considered. The use of biological measures is a rarely used method in RPM but research, as seen from the study by Patel et al [65], is needed to develop cost-effective tools for predicting AECOPD.

### Limitations

This review has some limitations. The number of studies initially identified in searches is relatively small and limited to those full and freely available in English. This is due to a combination of stringent search terms to identify RCT and machine learning papers and the strict inclusion and exclusion criteria. This may influence our confidence in drawing conclusions regarding the efficacy or accuracy of RPM and machine learning in COPD or, international variations, or those not freely available cannot be reported. However, clearly defined criteria are necessary to minimize bias and subjectivity in the search and enable reproducibility and rigor that add validity to a systematic review. This study did not include a formal risk of bias assessment. However, evaluating the studies using CASP checklists and ranking them should highlight the quality of the studies included in this dual systematic review.

### Recommendations

The methodologies for exacerbation intervention using RPM exhibit inconsistency, potentially contributing to the variability in the success of outcomes. The current landscape is hindered by inadequate evidence and the substantial resource burden associated with clinical oversight of remotely monitored indices. Therefore, it becomes apparent that a shift away from conventional intervention methods is warranted, with a compelling case for exploring the integration of machine learning approaches as a more pragmatic and potentially effective alternative.

While the literature emphasizes the accuracy of machine learning in AECOPD prediction, demonstrating the clinical use of these approaches requires evidence from RCT and real-world studies, both of which are currently lacking.

Physiological and functional indices, such as SpO_2_, RR, HR, weight, FEV1, and PEF, are commonly found in the literature and have the potential to serve as robust predictors of AECOPD when integrated with machine learning. Exploring the inclusion of PA as a predictive index is justified, and the incorporation of PRO, particularly through clinically validated questionnaires, is strongly recommended.

There is a lack of research into patient factors, there is a need to study the adoption of RPM in the long term and to develop an understanding of the burden of daily or weekly RPM and potential solutions to overcome this burden. Active engagement with patient communities is required to understand their needs, ensuring optimal responses and the best possible outcomes.

### Conclusion

RPM has yet to consistently prove a successful intervention in AECOPD, facing significant challenges with accuracy in predicting and identifying exacerbations, resource-intensive processes, and scalability limitations. Although machine learning demonstrates the potential for high accuracy for AECOPD, its clinical use is still to be validated through RCTs. Considering the heterogeneity of COPD, a one-size-fits-all approach may not be suitable. Leveraging machine learning, an individualized approach tailored to each patient’s unique data holds the potential to enhance patient outcomes in AECOPD.

## Appendix

Multimedia Appendix 1 PRISMA (Preferred Reporting Items for Systematic Reviews and Meta-Analyses) checklist.

Multimedia Appendix 2 Search methods and strategy.

Multimedia Appendix 3 A detailed breakdown of the study characteristics, method of intervention delivery, and outcomes.

## Abbreviations

AECOPD: acute exacerbations of chronic obstructive pulmonary disease
AUROC: area under the receiver operating characteristic curve
CASP: Critical Appraisal Skills Programme
CAT: chronic obstructive pulmonary disease assessment test
COPD: chronic obstructive pulmonary disease
CRP: C-reactive protein
ED: emergency department
FEV1: forced expiratory volume in 1 second
HR: heart rate
HRQoL: health-related quality of life
PA: physical activity
PR: pulse rate
PRISMA: Preferred Reporting Items for Systematic Reviews and Meta-Analyses
PRO: patient-reported outcomes
RCT: randomized controlled trial
RNN: recurrent neural network
RPM: remote patient monitoring
RR: respiratory rate
SpO_2_: oxygen saturation
WISDOM: Withdrawal of Inhaled Steroids during Optimized Bronchodilator Management
