# Supplementary material for: Remote Patient Monitoring and Machine Learning in Acute Exacerbations of Chronic Obstructive Pulmonary Disease: Dual Systematic Literature Review and Narrative Synthesis
Source: J Med Internet Res. 2024 Sep 9;26:e52143. doi: 10.2196/52143 (PMC11420610; doi:10.2196/52143)
Supplement: Multimedia Appendix 2 [file jmir_v26i1e52143_app2.docx]

### Search methods and strategy

We show the search strategy for a) SCOPUS remote patient monitoring, b) Web of Science remote patient monitoring, c) SCOPUS remote patient monitoring and modelling, and d) Web of Science remote patient monitoring and modelling.

1. TITLE-ABS-KEY ( ( copd OR ”chronic obstructive pulmonary disease” OR ”chronic obstructive lung disease” ) AND ( telemedicine OR telemonitoring OR ”remote patient monitoring” OR ”remote monitoring” OR ”continuous monitoring” OR ”real-time monitoring” OR telehealth OR ”mobile health” OR mhealth OR ”digital health” ) AND ( “RCT” OR “randomised controlled trial” OR “randomised trial” OR “randomised clinical trial” OR “clinical trial”) AND ( admission* OR readmission* OR exacerbation* OR ”quality of life”) AND NOT (feasibility OR pilot OR review OR rehab* ))
2. TS=(copd OR chronic obstructive pulmonary disease OR chronic obstructive lung disease) AND TS=(telemedicine OR telemonitoring OR remote patient monitoring OR remote monitoring OR continuous monitoring OR

real-time monitoring OR telehealth OR mobile health OR mhealth OR digital health) AND TS=(RCT OR randomised controlled trial OR randomised trial OR randomised clinical trial OR clinical trial) AND TS=(admission* OR readmission* OR exacerbation* OR quality of life) NOT TS=( feasibility OR pilot OR review OR rehab*)

1. TITLE-ABS-KEY ( ( copd OR ”chronic obstructive pulmonary disease” OR ”chronic obstructive lung disease” ) AND ( telemedicine OR telemonitoring OR ”remote patient monitoring” OR ”remote monitoring” OR ”continuous monitoring” OR ”real-time monitoring” OR telehealth OR ”mobile health” OR mhealth OR ”digital health” ) AND ( prediction OR algorithm OR ”machine learning” OR ”deep learning” ) )
2. TS=(copd OR chronic obstructive pulmonary disease OR chronic obstructive lung disease) AND TS=(telecare OR telemedicine OR telemonitoring OR remote patient monitoring OR remote monitoring OR continuous monitoring OR real-time monitoring OR telehealth OR mobile health OR mhealth OR digital health) AND TS=(prediction OR algorithm OR machine learning OR deep learning)
