# Supplementary material for: Remote Patient Monitoring and Machine Learning in Acute Exacerbations of Chronic Obstructive Pulmonary Disease: Dual Systematic Literature Review and Narrative Synthesis
Source: J Med Internet Res. 2024 Sep 9;26:e52143. doi: 10.2196/52143 (PMC11420610; doi:10.2196/52143)
Supplement: Multimedia Appendix 3 [file jmir_v26i1e52143_app3.docx]

| Table S1: Summary characteristics of randomised controlled trials and remote patient monitoring in COPD. | | | | | | | | | | |
| --- | --- | --- | --- | --- | --- | --- | --- | --- | --- | --- |
| **1^st^ Author (year)** | **Study Length** | **No. of Participants** | | **Female %** | | **Mean Age (Years)** | | **Mean FEV_1_ (Predicted)** | | **RPM Indices** |
|  |  | **I** | **C** | **I** | **C** | **I** | **C** | **I** | **C** |  |
| Toledo (2006) [17] | 1 year | 67 | 90 | 2 | 3 | 71 | 72 | 42% | 42% | ECG, lung function, SpO2, BP, HR, and PRO |
| Koff (2009) [18] | 3 months | 20 | 20 | 50 | 55 | 67 | 65 | 34% | 31% | PRO (dyspnea, cough, sputum, fever), PA, depression, lung function, 6MWT, and SpO2 |
| Vitacca (2009) [19] | 1 year | 57 | 44 | 36 | 28 | 61 | 61 | 39% | 34% | PRO (dyspnea, cough, sputum, weight, fever, neurological status, ventilator use, and walk), SpO2, and HR |
| Halpin (2011) [20] | 4 months | 40 | 39 | 26 | 26 | 69 | 70 | 48% | 53% | PRO: breathlessness, cough, congestion, and fatigue |
| Dinesen *(2012)* [21] | 4 months | 57 | 48 | n/a | n/a | 68 | 68 | 0.9L | 0.9L | BP, PR, weight, SpO2, and lung function |
| De San Miguel (2013) [22] | 6 months | 36 | 35 | 61 | 43 | 71 | 74 | n/a | n/a | BP, weight, temperature, PR, SpO2 and general state of health |
| Jehn (2013) [23] | 9 months | 32 | 30 | 19 | 27 | 64 | 69 | 50% | 53% | CAT, lung function, and 6MWT (accelerometery) |
| Pedone (2013) [24] | 9 months | 50 | 49 | 28 | 57 | 74 | 75 | 53% | 55% | Wristband monitor (HR, PA, temperature, and galvanic skin response), and SpO2 |
| Pinnock (2013) [25] | 1 year | 128 | 128 | 59 | 51 | 75 | 68 | 45% | 40% | Medication self-report, SpO2, and upper respiratory infection, fever identification questionnaires and PRO (dyspnea, sputum purulence, sputum volume, cough, wheeze) |
| Sorknaes (2013) [26] | 6 months | 132 | 134 | 60 | 62 | 71 | 72 | 33% | 37% | PR, SpO2, and lung function |
| McDowell (2015) [27] | 6 months | 55 | 55 | 58 | 55 | 70 | 70 | 46% | 43% | BP, HR, SpO2 and PRO (dyspnea, cough, sputum, tiredness) |
| Ringbæk (2015) [28] | 6 months | 141 | 140 | 61 | 45 | 70 | 70 | 35% | 34% | Lung function, SpO2, weight, and PRO (dyspnea, sputum colour, volume, and purulence) |
| Cordova 2016 [29] | 2 years | 34 | 33 | 50 | 73 | 64 | 63 | 31% | 32% | Daily lung function and PRO (dyspnea, and sputum quantity, colour, and consistency, cough, wheeze, sore throat, nasal congestion, and high temperature) |
| Ho 2016 [30] | 6 months | 53 | 53 | 19 | 28 | 81 | 79 | 62% | 62% | SpO2, temperature, BP, and PRO (disease-related symptoms) |
| Vianello 2016 [31] | 1 year | 230 | 104 | 29 | 27 | 76 | 76 | 42% | 42% | HR and SpO2 |
| Farmer 2017 [32] | 1 year | 110 | 56 | 38 | 39 | 70 | 70 | 47% | 50% | PRO, SpO2, and HR |
| Lilholt 2017 [33] | 1 year | 258 | 316 | 46 | 55 | 68 | 70 | 49% | 50% | PRO (questions related to COPD exacerbations and symptoms), BP, HR, weight, and SpO2 |
| Rixon 2017 [34] | 1 year | 275 | 172 | 45 | 43 | 71 | 72 | n/a | n/a | SpO2, BP, weight, and questions about health |
| Kessler 2018 [35] | 1 year | 157 | 162 | 31 | 30 | 67 | 67 | 38% | 36% | Lung function, SpO2, and HR – participants on long-term oxygen therapy also had daily oxygen use and RR |
| Soriano 2018 [36] | 1 year | 115 | 114 | 22 | 18 | 71 | 71 | 34% | 32% | SpO2, BP, lung function, RR, and oxygen therapy compliance |
| Tupper 2018 [37] | 6 months | 141 | 140 | 61 | 45 | 70 | 69 | 35% | 34% | PRO (dyspnea, sputum colour, volume, and purulence), lung function, SpO2, and weight |
| Walker 2018 [38] | 9 months | 135 | 158 | 34 | 34 | 70 | 71 | 49% | 50% | Within-breath respiratory mechanical impedance using forced oscillation technique |
| Mínguez Clemente 2020 [39] | 6 months | 58 | 58 | 55 | 65 | 68 | 70 | 50% | 52% | ECG, SpO2, HR, BP, temperature, and RR |
| Sink 2020 [40] | 8 months | 83 | 85 | 54 | 53 | 64 | 62 | 64% | 63% | Breathing (better, same, worse) |
| Koff 2021 [41] | 9 months | 352 | 159 | 42 | 33 | 68 | 68 | 36% | 38% | PRO, SpO2, lung function, 6MWT (pedometer), and post-exertion SpO2 |
| Rassouli 2021 [42] | 1 year | 168 | 168 | 35 | 35 | 67 | 67 | 51% | 51% | PRO (dyspnea, sputum volume, sputum colour, cough, fever, emergency medication) |
| Anderson 2023 [43] | 6 months | 110 | 112 | 65 | 57 | 70 | 71 | 35% | 38% | SpO2, HR, lung function, weight, and PRO (dyspnea, cough, sputum volume, and colour) |
| Køpfli 2023 [44] | 6 months | 101 | 97 | 65 | 57 | 69 | 70 | 39% | 40% | SpO2, HR, lung function, weight, and PRO (dyspnea, cough, sputum volume and sputum colour) |
| **Abbreviations:** I = intervention, C = control, ECG = electrocardiogram, SpO2 = oxygen saturation, BP = blood pressure, HR = heart rate, PRO = patient reported outcomes, FEV_1_ = forced expiratory volume in 1 second, 6MWT = 6-minute walking time, PR = pulse rate, PA = physical activity, PEF = peak expiratory flow, RR = respiratory rate. | | | | | | | | | | |

| Table S2: CASP RCT checklist ranking of randomised controlled trials of remote patient monitoring in COPD. | | |
| --- | --- | --- |
| **1^st^ Author** | **Intervention** | **Effect Size** |
| **Strongest Evidence** | | |
| Pinnock [25] | Patient data was monitored daily by a supporting clinical team (specialist respiratory team, a nurse specialist, or a trained call handler) and alerts were generated for no submission or a high symptom score resulting in action by the monitoring clinician. | There was no significant difference in the number of days to admission, the mean number of admissions, and the duration of admissions, or HRQL. |
| Sink [40] | A daily message from the EpxCOPD system inquires “Are you breathing better than, worse than, or the same as yesterday?”. Responding “worse” triggers an alert to an assigned resident clinic. The resident would contact the participant and then counsel on how to return to normal breathing or order an intervention. | Time-to-hospitalisation was significantly longer in the intervention, HR=2.36 (95% CI=1.02–5.45, p=0.0443). The number needed to treat was 8.62. |
| **Strong Evidence** | | |
| Køpfli [44] | The measures were sent every weekday for the first month, and three times a week during the last 5. A threshold was set for each measurement in each participant. Based on the patient’s reported measurements and symptoms there were three categories: green (stable), yellow (change in symptoms), and red (change in physiological measurement). The respiratory nurses evaluated the data and with a respiratory physician determined additional treatment or follow-up. | There was no significant difference in SGRQ, HADS-Anxiety, or HADS-depression. No difference was seen at 12–24 months follow-up. |
| Mínguez Clemente [39] | Patients transmitted data twice daily. Thresholds were set for the measures. Deviations from the thresholds generated an alert in the form of a text message sent to a physician’s telephone. The physician could contact the participant for intervention. | There was no significant difference in baseline characteristics, time until first exacerbation, the number of exacerbations or costs. There was a significant decrease in the number of visits observed in the intervention compared to the control, 3.8±1 vs 5.1±2 (p=0.001). |
| Walker [38] | An algorithm generated respiratory alerts if a trend of worsening was detected (increase of inspiratory resistance, difference between inspiratory and expiratory reactance, or decrease of inspiratory reactance). The alert triggered contact with the study nurse to determine intervention. | There was no significant difference in time-to-first-hospitalisation, EQ-5D score, antibiotic prescriptions, hospitalisation rate, or questionnaire scores. In an exploratory analysis, the intervention was associated with fewer repeat hospitalisations (-54%, p=0.017) |
| Lilholt [33] | Data was sent to healthcare personnel, if the data adversely deviated from normal threshold values the patients were contacted. Patients were also contacted if the measurements were not carried out as agreed or the measurements were not received as expected. | There was no significant difference in HRQL as assessed by the PCS and MCS. |
| Vianello [31] | Operators reviewed the patient data and if values were outside of the patient’s “normal” range, the patient was contacted for a second measurement. If also outside the normal range clinical staff were alerted and would contact the patient to check for worsening and possible intervention. | There was no significant difference in HRQL as assessed by PCS, MCS, and HADS. Hospital admission rates did not significantly differ between groups. However, the readmission rate for AECOPD was significantly lower in the intervention group, IRR=0.43 (95% CI 0.19–0.98, p=0.01). |
| Ho [30] | Data was processed according to a predefined algorithm. If the algorithm detected risk of an exacerbation a warning was generated and sent to nurses and attending pulmonary physicians. The patient’s data was then assessed which could result in referral to the clinic or emergency department. | At 6 months, time to first readmission was significantly increased (p=0.026) and COPD-related readmission was significantly lower, HR=0.42 (95% CI=0.19–0.92). |
| Pedone [24] | Each parameter was measured every three hours and was evaluated every day by a respiratory physician. Alerts were generated when a measurement was outside the predefined range causing the physician to contact the patient for appropriate management. | In the intervention, the occurrence of respiratory events was 28 per 100 person-years, compared to 42 per 100 in the control, IRR=0.67 (95% CI 0.32 – 1.36). Hospital admission rates were 13 per 100 person-years in the intervention and 20 per 100 in the control, IRR=0.66 (95% CI 0.21 – 1.86). |
| Cordova [29] | A symptom algorithm detected changes from initial participant symptom values. If the symptom score generated by the algorithm reached or exceeded the predetermined threshold participants contacted a number that was available 24h/day, 7 days a week, staffed by nurses and pulmonologists. Exacerbations were treated according to the GOLD guidelines. | There was no significant difference in hospitalisation rates, hospital duration, or mortality. The intervention group’s PEF, BDS, DASI, and SF-36 significantly improved and were sustained for up to 24 months but were unchanged in the control. |
| **Moderate Evidence** | | |
| Farmer [32] | An initial 6-week period of monitoring created a threshold for the participant safety alert. Participants continued to input their data daily. A respiratory clinician reviewed a summary of the data twice weekly. If data were not received or there were safety alerts, the participant record was accessed for review. If there was a clinically important change in the data, then the patient was contacted. | There was no significant difference in SGRQ or hospital admission. The intervention had better health status (EQ-5D), 0.076 (95% CI 0.008-0.14, p=0.03). The intervention had 4 visits to GP compared to 5.5 in the control (p=0.06) and fewer nurse contacts, 1.5 compared to 2.5 (p=0.03). |
| McDowell [27] | A trend report was created to set normal limits. Daily data was reviewed by a nurse who compared it to the set limits. An alert was generated if clinical and symptom observations were outside the limits. A nurse would then contact the patient, repeat the monitoring, and would escalate based on the results. | There was no significant difference in EQ-5D scores, HADS depression scores, GP activity, ED presentations, hospital admissions or exacerbations. However, SGRQ scores were significantly lower in the Intervention, -5.75 (95% CI 2.32-9.18, p=0.001) and HADS anxiety score were significantly lower, -1.66 (95% CI 0.37-2.95, p=0.01) |
| De San Miguel [22] | The data was monitored daily by a nurse. Any deviations outside an individual’s normal parameters (specified by their GP or specialist) trigger an alert. The nurse would contact the participant and provide advice or recommend an appointment to visit the GP. | There was no significant difference in ED presentations, hospital admissions, or length of stay. |
| Anderson [43] | The monitoring took place every weekday for the first month and then three times a week. An algorithm generated alerts if a trend of worsening was detected: green (stable), yellow (one or more PRO indicated clinical worsening), and red alert (one or more measurements were abnormal). Yellow or red alerts triggered contact to the participant by the respiratory nurse. Treatment, frequent monitoring, outpatient appointments, contact with the patient´s GP or hospitalisation could be started. | There was no significant difference in percentage of patients who had an AECOPD hospitalisation or time to first hospitalisation. However, the number of hospitalisations was 66 in the control group and 42 in the intervention, IRR=1.42 (95% CI=1.04–1.95, p=0.03). |
| Rassouli [42] | Participants answered questions every morning. Answers were coded: not answered (grey), had answered zero or one question with ‘yes’ (green), had answered two or more questions with ‘yes’, but was green the previous day (yellow), or had answered two or more questions with ‘yes’ and had a yellow or red the previous day (red). Red indicated a possible AECOPD and resulted in participants being contacted by a physician for intervention. | There was no significant difference in the ED presentations, hospitalisation rate, number of AECOPD, or days in hospital due to AECOPD. However, the CAT score increase in the intervention vs. control was 1.8 vs. 3.6 points/year (p=0.0015). |
| Soriano [36] | Reference values were generated in the first four days. The monitoring centre received the information as Red: “clinical alert” – measurements exceeded reference values, Yellow: “Technical Alert” – measurements missing, or Green: The measurements had been made and found to be within the reference. The “clinical alerts” were confirmed by a clinical questionnaire which resulted in notifying the relevant pulmonologist/local coordinator. | There was no significant difference in the proportion of participants who had an AECOPD or the number of all-cause deaths. There was a trend towards a shorter duration of hospitalisation and intensive care in the intervention compared though this was non-significant. |
| Dinesen [21] | A healthcare professional such as a GP, nurse, or doctor at a healthcare centre or hospital could assess and monitor the patient’s data and provide advice to the patient. | The intervention group had a reduced mean hospital admission rate compared to the control, 0.49 vs 1.17 (p=0.041). However, there was no significant difference in the length or cost of admission. |
| Halpin [20] | An increase of over 2 points in the mean of the summed raw daily scores on the last two days compared to the previous five days resulted in flagging the patient data. Subsequently, an alert call to the patient would be made. | There was no significant difference in exacerbation frequency, duration, or severity, or SGRQ scores. Exacerbation frequency was greater on predicted high-risk vs non-high-risk days. |
| Toledo [17] | Clinical specialists directly monitored patient data. | There were significantly more patients that were not readmitted in the intervention vs control, 51.7% vs 33.3% (p=0.04), respectively. |
| Vitacca [19] | At baseline, a nurse recorded indices. If there was a score variation greater than 3 points from baseline, the nurse contacted a pulmonologist for consultation. | The intervention group experienced fewer hospitalisations (-36%, p=0.018), urgent GP calls (-65%, p=0.013), and AECOPD (-71%, p=0.0003). |
| **Limited Evidence** | | |
| Jehn [23] | Patient data was transmitted directly to the study centre which was reviewed by a physician daily. | There was a significant reduction in exacerbations (7 vs. 22, p=0.012), improvement in CAT score (-2.9±4.5, p=0.004 vs +4.4±5.7, p=0.013), and increase in 6MWT distance (+87.0 ± 65.7 metres, p=0.006 vs +23.9 ± 70.3 metres, p=0.23), in the intervention vs control. |
| Sorknaes [26] | Patients were monitored for 7 days following admission and had daily remote consultations by video calls with a nurse who collected the patient’s measures. The nurse could organise rapid treatment in consultation with a physician, GP and/or the home care system if needed. | There was no significant difference in the total mean number of hospital readmissions, mortality, time to readmission, or hospital readmission days. |
| Ringbæk [28] | Patient data was transferred to a call centre at each participant’s local hospital and was automatically categorised and prioritised. The call centres were staffed by a trained respiratory nurse who could confer the patient’s data with a specialist in respiratory medicine at the hospital if values were alarming. | There was no significant difference in hospital admissions, time to first admission, or all-cause hospital admission. However, the intervention had significantly lower visitation to the outpatient clinic (0.22 vs 0.99, p=0.001) and had more exacerbations treated at home with rescue medication (58.2% vs 37.1%, p=0.001) |
| Koff [41] | Measurements were taken every weekday and were analysed by predetermined algorithms, and categorised into green (stable), yellow (caution), and red (possible decline in health status). Coordinators contacted participants with red flags and resolved clinical problems directly or by reaching the participant’s primary care provider. | The intervention group saw improvements in SGRQ by 7–9 units (p=0.001), increased 6MWT distance by 40m (p=0.001), reduced urgent office visits by 76 visits per 100 participants (p=0.0001), and had a reduction in symptoms, BODE index and oxygen titration (p=<0.05) when compared with the control group. However, mortality, hospitalisation, intensive care visits, ED presentation, and length of stay were not significantly different between groups. |
| Tupper [37] | Measurements were performed every week and were transferred to a call centre at each participant’s local hospital and automatically categorised and prioritised (green, yellow, or red). Concerning measurements resulted in a phone call from a specialist nurse either by phone or video consultation and possibly conferred with a respiratory specialist. | At 6 months, the 15D significantly improved (0.016, p=0.03) in the intervention group (compared to baseline), whilst there was no improvement in the control group (-0.003, p=0.68). However, there was no statistically significant change in the CAT score. |
| Koff [18] | A registered respiratory therapist monitored data and contacted patients if red flags were raised on changes in measures. | The SGRQ improved (decreased) by 10.3 in the intervention vs. 0.6 in the control (p=0.018). |
| Kessler [35] | Information was transmitted by patients using a telephone-based questionnaire once per week, and each day of symptom worsening. An e-health platform enabled patient follow-up by case managers for early detection of exacerbations. Information was transmitted to hospital physicians via the e-platform to intervene when necessary. | There was no significant difference in the number of participants with exacerbations, exacerbation frequencies, time to first exacerbation, and all-cause hospitalisation days per year. However, the intervention had significantly fewer acute care hospitalisation days per year (−8.3 days, 95%, p=0.047), a lower BODE index (-0.5, p=0.01) and a lower mortality rate (1.9% vs 14.2%, p=0.001). |
| Rixon [34] | Data was reviewed by healthcare professionals at a monitoring centre. Review of data would result in no response, requesting another reading, contacting the participant or their carer, a home visit by their community nurse or referral to a health service. | There was no significant difference for improvement in dyspnea, fatigue, SF-12, EQ5D, anxiety, and depression. |
| **Abbreviations:** HRQL = health-related quality of life, CI = confidence interval, HR = hazard ratio, SGRQ = St Georges respiratory questionnaire, HADS = hospital anxiety and depression scale, PCS = physical component summary, MCS = mental component summary, IRR = incidence rate ratio, DASI = DUKE activity status index, Borg dyspnea scale, SF-36 = 36-item short form health survey, GP = general practitioner, CAT = COPD assessment test, BODE index = Body mass index, airflow Obstruction, Dyspnea, and Exercise capacity, ED = emergency department, AECOPD = acute exacerbation of COPD, 6MWT = 6 minute walking test, 15D = 15- dimensional health-related quality of life measure, SF-12 = 12-item short form health survey. | | |

| Table S3: Summary characteristics of machine learning and remote patient monitoring studies in COPD for exacerbation prediction. | | | | | | |
| --- | --- | --- | --- | --- | --- | --- |
| **1^st^ Author (year)** | **Observation period** | **No. of Participants** | **Female %** | **Mean Age (Years)** | **Disease Severity** | **RPM Indices** |
| Jensen (2012) [45] | 4 months | 57 | 60 | 68 | FEV_1_ = 0.9 L | Weight, BP, SpO2 and, lung function |
| Heijden (2013) [46] | 6 months | 5 | n/a | n/a | n/a | Lung function, SpO2, and PRO (dyspnea, sputum production, sputum colour, cough, wheezing, activities, malaise, fever) |
| Bellos (2013) [47] | n/a | 30 | n/a | n/a | n/a | ECG, RR, steps, standing and lying time, cough, snoring, temperature, BP, SpO2, lung function, blood glucose, weight, PRO (dyspnea, cough, sputum), and demographics (lifestyle, mental status) |
| Fernández-Granero (2014) [48] | 6 months | 16 | 71 | 70 | GOLD COPD stage > A | PRO (general health status, cough, phlegm, dyspnea, sleep, cold symptoms, lung sounds and coordination test) |
| Shah (2014) [49] | 6 months | 18 | 50 | 71 | SpO2 = 95% | PRO (general health status, breathlessness, wheeze, cough, sputum colour, presence of cold, sleep breathing), SpO2, HR, RR, and PPG waveform |
| Fernández-Granero (2015) [50] | 6 months | 16 | 71 | 70 | GOLD COPD stage = C & D | PRO (breathlessness, cough, sputum, chest symptoms, difficulty bringing up sputum, fatigue, sleep disturbance, and health anxiety) |
| Fernández-Granero (2015) [51] | 6 months | 16 | 71 | 70 | GOLD COPD stage = C & D | Respiratory sounds through a respiratory sensor embedded in a self-tailored housing. |
| Mohktar (2015) [52] | 1 year | 21 | 71 | 71 | GOLD COPD stage= B-D | Lung function, SpO2, RR, HR, temperature, and weight |
| Sanchez-Morillo (2015) [53] | 6 months | 16 | 71 | 70 | GOLD COPD stage = C & D | PRO (general health status, cough, phlegm, dyspnea, sleep, cold symptoms, lung sounds, and coordination test) |
| Christian Riis (2016) [54] | 2 years | 108 | 52 | 70 | FEV_1_ = 0.76 L | PRO, BP, SpO2, and PR |
| Shah (2017) [55] | 1 year | 110 | 38 | 70 | FEV_1_ = 47% | PR, RR, and SpO2 |
| Fernández-Granero (2018) [56] | 6 months | 16 | 71 | 70 | GOLD COPD stage = C & D | Respiratory sounds were recorded daily with an electronic sensor |
| Kronborg (2018) [57] | 1 year | 57 | 56 | 69 | n/a | PRO (symptoms, cough, mucus, shortness of breath, inhaler use, antibiotic use), SpO2, PR, weight, and BP |
| Nunavath (2018) [58] | 2 years | 94 | 49 | 69 | FEV_1_ = 38% | Symptom-specific questionnaire, SpO2, and results of automatically generated health status overview |
| Orchard (2018) [59] | 1 year | 135 | 40 | 70 | n/a | Symptoms, physiological measures, and medication data, with baseline demography, COPD severity, quality of life, and hospital admissions linked with meteorological data |
| *Boer *et al.* (2018-19) [60, 61] | 1 year | I = 43  C = 44 | I = 42  C = 34 | I = 69  C = 66 | FEV_1_  I = 53%  C = 52 % | PRO (12 yes-or-no questions), SpO2, lung function |
| Jin (2018) [62] | 1 month | 22 | 18 | 76 | n/a | Non-invasive ventilator: airflow, pressure and SpO2 |
| Iadanza (2020) [63] | n/a | 424 | n/a | n/a | n/a | FVC, SVC, FEV_1_/FVC ratio, FEV_1_/SVC ratio, forced expired flow at 25–75\%, PEF, FVC, TLC, RV, FRC, ERV, DLCO, VA, and DLCO/VA |
| Kronborg (2021) [64] | 2 years | 9 | n/a | 70 | mMRC dyspnea = 3.8 | SpO2, PR, and BP |
| Patel (2021) [65] | 6 months | 90 | 50 | 69 | FEV_1_ = 50% | PRO, lung function, CRP (finger-prick testing) |
| Wu (2021) [66] | 4 months | 67 | 12 | 67 | FEV_1_ (30-79%) = 66% | Environmental (temperature, humidity, particulates), HR, steps, deep/light/REM sleep time, calories, PRO (CAT, mMRC dyspnea, quality of life) |
| Chmiel (2022) [67] | 2 years | 2374 | n/a | 60-79 | n/a | PRO (CAT score, symptom score) demographics (Age, gender, smoking years, smoking status) |
| Wu (2022) [68] | 2 years | 106 | n/a | n/a | n/a | Air pollution, PA, PRO, and lung function |
| Abbreviations: BP = blood pressure, SpO2 = oxygen saturation, FEV_1_ = forced expiratory volume in 1 second, PRO = patient reported outcome, ECG = electrocardiogram, RR = respiratory rate, GOLD = Global Initiative for Chronic Obstructive Lung Disease, HR = heart rate, PPG = photoplethysmogram, PR = pulse rate, I = intervention, C = control, FVC = forced vital capacity, SVC = slow vital capacity, PEF = peak expiratory flow, RV = residual volume, FRC = functional residual capacity, ERV = expiratory reserve volume, DLCO = diffusing capacity of the lungs for carbon monoxide, VA = alveolar volume, CRP = C-reactive protein, REM = rapid eye movement, CAT = COPD assessment test, mMRC dyspnea = modified medical research council dyspnea scale, PA = physical activity.  *Boer [60] is a model development study, Boer [61] is an RCT. Patient data is from the RCT. | | | | | | |

| Table S4: Narrative synthesis and evidence ranking based on CASP cohort checklist of studies using machine learning and remote patient monitoring in COPD for exacerbation prediction. | | |
| --- | --- | --- |
| **1^st^ Author** | **Model Development** | **Model Accuracy** |
| **Strongest Evidence** | | |
| Mohktar [52] | Patient data was monitored daily and was used to train a CART model to predict if patients are at high risk or low risk of exacerbation. | The CART model has an accuracy of 71.8%, specificity of 80.4%, and 61.1% sensitivity |
| Orchard [59] | Data from the Telescot COPD telemonitoring program was used to construct predictive models fitted to training sets of patient data using MTNN. | The MTNN based on 57,150 episodes has an AUROC of 0.74 (95% CI 0.67-0.80), a specificity of 60% and a sensitivity of 80%. |
| Nunavath [58] | This study used ANN; FFNN for the classification of COPD patient's health category, and LSTM for early prediction of COPD exacerbations and subsequent triage. | The FFNN model can reproduce diagnosed health conditions with an accuracy of 92.86% and the LSTM model was able to predict COPD patients’ health conditions one-day with an accuracy of 84.12%, a specificity of 6.06%, and a sensitivity of 99.41%. |
| *Boer [60, 61] | The intervention used the smart tool ACCESS which uses a built-in Bayesian network decision model, the mHealth tool offers advice like adjusting bronchodilator use, practicing breathing and coughing techniques, managing energy distribution, reaching out to a healthcare professional, or measuring again tomorrow. | In the initial validation study ACCESS has a sensitivity of 97.4%, a specificity of 65.6%, and a PPV and NPV of 13.4% and 99.8, respectively. In the RCT there was not a statistically significant difference between the intervention and the control in exacerbation-free weeks, health status, self-management behaviour, or health care utilisation |
| **Strong Evidence** | | |
| Chmiel [67] | patent-entered self-management app data from the myCOPD app was used to generate AECOPD prediction models using LR and RF. | The RF with Youden’s J statistic as threshold achieved an AUROC of 0.727, a sensitivity of 0.755, and a specificity of 0.629. |
| Patel [65] | Patients performed a daily wellbeing assessment and weekly spirometry, a decision tree could prompt further testing of spirometry and/or CRP test, and the exacerbation prediction model was trained on a 2-week learning phase. | Compared to the 6 months pre-intervention, hospitalisations were reduced by 98% (90 vs 2, p=0.001), the model has a sensitivity of 97.9%, a specificity of 84.0%, and a PPV and NPV of 38.4% and 99.8%, respectively. |
| Wu [66] | A DT, RF, KNN clustering, LDA, AdaBoost and DNN were used to generate the AECOPD prediction models. | The DNN model performed the best at predicting AECOPD within 7 days with an f1 of 0.923, an accuracy of 92.1%, a sensitivity of 90.4%, a specificity of 94% and an AUROC 0.964. |
| Shah [55] | This retrospective cohort study using data from the EDGE clinical trial used participant data to train a LR classifier. | Tenfold cross-validation was repeated 10 times to evaluate the model which has an AUROC of 0.682%. The model has an 80% sensitivity and 36% specificity, or 60% sensitivity and 68% specificity for AECOPD. |
| Christian Riis [54] | Patients either did daily or weekly measurements depending on disease severity. KNN was used to develop the prediction model. | A leave-one-out cross-validation was used to evaluate, and the best model has a sensitivity of 73%, a specificity of 74%, a PPV of 69% and an NPV of 78% for AECOPD. |
| Kronberg [64] | The one-layer model is developed using extracted features. Subsequently, a two-layer model is constructed by leveraging features extracted from the probability estimates of exacerbation within the one-layer model. These features were used to train an exacerbation prediction model using LR, LDA, SVM, KNN, naive Bayes, CART, and RF. | SVM with radial basis function on the two-layer model performed the best with an AUROC of 0.95 and a sensitivity of 0.94. |
| Wu [68] | Through the feature importance map and SHAP module, then applying backward elimination cost-effective features were selected and used to train an RF model. | The cost-effective RF model performed the best at predicting AECOPD within 7 days with an accuracy of 88.6%, a sensitivity of 77.8%, a specificity of 94.9% and an f1 of 0.833. |
| Kronborg [57] | This was a retrospective validation study using data collected from the TeleCare North trial using LR to generate exacerbation prediction models. | The best-performing model achieved an AUROC of 0.74 for AECOPD which was based on two-fold patient-dependent cross-validation. |
| Fernández-Granero [51] | This was a retrospective model development study using data from a pilot study and PCA and SVM. | SVM classifier achieved a sensitivity of 73.76%, a specificity of 97.67%, a PPV of 84.66% and an NPV of 95.53% for predicting AECOPD. 75.8% exacerbations were detected early 5±1.9 days in advance of intervention for AECOPD. |
| **Moderate Evidence** | | |
| Fernández-Granero [48] | Recorded data were used to train and validate a PNN model to predict AECOPD. | The PNN model forecasted COPD exacerbations with a margin of 4.8±1.8 days. The PPV was 91.67%. |
| Shah [49] | The data from a pilot study pilot enabled the development of a model through multivariate novelty detection using Parzen windows. | The model achieved an AUROC of 0.91 for AECOPD. |
| Fernández-Granero [56] | A retrospective cohort study to enable an automatic prediction of symptom-based exacerbations, recorded data were used to train and validate an RF classifier. | With a 4.4 days margin before the onset of AECOPD, the sensitivity of the model is 78.1%, the specificity is 95.9%, and the PPV is 94.1%. |
| Sanchez-Morillo [53] | K-means clustering with five-fold cross-validation to select the R-value was used to generate an exacerbation prediction model. | Using tenfold cross-validation to evaluate, the model achieved an accuracy of 84.7%, a sensitivity of 74.6%, a specificity of 89.7%, PPV 78.5%, NPV 87.6% for AECOPD. |
| Fernández-Granero [50] | A retrospective study where participants answered questions daily and a PNN was used for classification. | Ten-cross validation was used to evaluate the model which for a symptom-based definition has an accuracy of 88.3%, a sensitivity of 80.5%, a specificity of 94.34%, a PPV of 91.67%, and a negative-predictive value NPV of 86.21% and can detect AECOPD on average 4.8 ± 1.8 days to the event. |
| **Limited Evidence** | | |
| Iadanza [63] | A neural network, SVM and C5.0 were used to generate the AECOPD prediction models. | C5.0 performed best across mild, moderate, and severe COPD. The model performed best in severe COPD achieving a sensitivity of 98.9%, a specificity of 96.2%, and an accuracy of 97.4% for AECOPD. |
| Jin [62] | SVM, RF and LDA were used to generate exacerbation prediction models. | To evaluate the models, they used five-fold cross-validation. The best-performing model is the LDA model with an accuracy of 74.5%, a sensitivity of 77.6%, and a specificity of 42.9%. |
| Jensen [45] | This was a retrospective cohort study to train and validate models generated using an LDA algorithm on data taken from the TELEKAT trial. | Evaluating the model in a leave-one-out cross-validation, the model has a sensitivity of 70%, a specificity of 95%, and an AUROC of 0.73 for predicting AECOPD. |
| Heijden [46] | A Bayesian network model was used to predict the risk of exacerbation. Patient data were collected weekly if the patient is at low risk of exacerbation, the check-in becomes daily with increasing risk. | The model performance on a different data set includes an AUROC of 0.87, a sensitivity of 0.88, a false-positive rate of 0.2 and an accuracy of 0.81 for predicting AECOPD. |
| Bellos [47] | Clinicians labelled the severity of the patient's situation to make the classes. A stratified tenfold cross-validation was used to randomise the records and split the available instances into training and testing datasets. The hybrid models were generated using correlation-based feature subset selection and a hybrid classification system combining RF, SVM, and a rule-based system. | The hybrid classifier achieved an accuracy of 94% for current disease severity estimation, which can be used to recognise an abnormal health episode or an AECOPD. |
| **Abbreviations:** AUROC = area under the receiver operating characteristic, PPV = positive predictive value, NPV = negative predictive value, CART = classification and regression tree, MTNN = multi-task neural network, ANN = artificial neural network, FFNN = feed forward neural network, LSTM = long short term memory, LR = logistic regression, RF = random forest, CRP = C-reactive protein, DT = decision tree, KNN = k-nearest neighbours, LDA = linear discriminant analysis, AdaBoost = adaptive boosting, DNN = deep neural network, SVM = support vector machine, PCA = principal component analysis, AECOPD = acute exacerbation of COPD.  *Boer [60] is a model development study, Boer [61] is an RCT. Boer [61] was assessed using CASP RCT checklist and ranked as such. | | |
